# A comparative study of nemertean complete mitochondrial genomes, including two new ones for *Nectonemertes *cf. *mirabilis *and *Zygeupolia rubens*, may elucidate the fundamental pattern for the phylum Nemertea

**DOI:** 10.1186/1471-2164-13-139

**Published:** 2012-04-17

**Authors:** Hai-Xia Chen, Shi-Chun Sun, Per Sundberg, Wei-Cheng Ren, Jon L Norenburg

**Affiliations:** 1 Department of Biological and Environmental Sciences, University of Gothenburg, PO Box 463, SE-405 30 Gothenburg, Sweden; 2 Institute of Evolution & Marine Biodiversity, Ocean University of China, 5 Yushan Road, Qingdao 266003, China; 3 Department of Rheumatology and Inflammation Research, Sahlgrenska Academy, University of Gothenburg, PO Box 480, SE-405 30, Sweden; 4 Department of Invertebrate Zoology, National Museum of Natural History, Smithsonian Institution, Washington, DC 20560-0163, USA

**Keywords:** MtDNA, Nemertea, *Nectonemertes mirabilis*, *Zygeupolia rubens*, Phylogeny, Gene rearrangement

## Abstract

**Background:**

The mitochondrial genome is important for studying genome evolution as well as reconstructing the phylogeny of organisms. Complete mitochondrial genome sequences have been reported for more than 2200 metazoans, mainly vertebrates and arthropods. To date, from a total of about 1275 described nemertean species, only three complete and two partial mitochondrial DNA sequences from nemerteans have been published. Here, we report the entire mitochondrial genomes for two more nemertean species: *Nectonemertes *cf. *mirabilis *and *Zygeupolia rubens*.

**Results:**

The sizes of the entire mitochondrial genomes are 15365 bp for *N*. cf. *mirabilis *and 15513 bp for *Z. rubens*. Each circular genome contains 37 genes and an AT-rich non-coding region, and overall nucleotide composition is AT-rich. In both species, there is significant strand asymmetry in the distribution of nucleotides, with the coding strand being richer in T than A and in G than C. The AT-rich non-coding regions of the two genomes have some repeat sequences and stem-loop structures, both of which may be associated with the initiation of replication or transcription. The 22 tRNAs show variable substitution patterns in nemerteans, with higher sequence conservation in genes located on the H strand. Gene arrangement of *N*. cf. *mirabilis *is identical to that of *Paranemertes *cf. *peregrina*, both of which are Hoplonemertea, while that of *Z. rubens *is the same as in *Lineus viridis*, both of which are Heteronemertea. Comparison of the gene arrangements and phylogenomic analysis based on concatenated nucleotide sequences of the 12 mitochondrial protein-coding genes revealed that species with closer relationships share more identical gene blocks.

**Conclusion:**

The two new mitochondrial genomes share many features, including gene contents, with other known nemertean mitochondrial genomes. The tRNA families display a composite substitution pathway. Gene order comparison to the proposed ground pattern of Bilateria and some lophotrochozoans suggests that the nemertean ancestral mitochondrial gene order most closely resembles the heteronemertean type. Phylogenetic analysis proposes a sister-group relationship between Hetero- and Hoplonemertea, which supports one of two recent alternative hypotheses of nemertean phylogeny.

## Background

Knowledge of mitochondrial genomes is important for many scientific disciplines [1,2] and the relative arrangement of mitochondrial genes has been effective for studying phylogenetic relationships [3,4]. However, current knowledge of mtDNAs is uneven, and sequences available in GenBank are predominantly from vertebrate taxa. There are about 1275 described species [5] of nemerteans (ribbon worms, phylum Nemertea); these are mainly marine but terrestrial and freshwater species also are known. To date, complete mitochondrial genomes have been published for only three species in the phylum, *Cephalothrix hongkongiensis *(Palaeonemertea) [reported as *Cephalothrix simula *in [6]], *Lineus viridis *(Heteronemertea) [7], and *Paranemertes *cf. *peregrina *(Hoplonemertea)[8]. Nearly complete sequences exist for the palaeonemerteans *Cephalothrix *sp. [8] and *Cephalothrix rufifrons *[9]. Thus, current genomic knowledge of nemerteans is scant and taxon diversity is poorly sampled. In this study, we sequenced the complete mitochondrial genomes of two nemertean species, *Nectonemertes *cf. *mirabilis *(Hoplonemertea: Polystilifera) and *Zygeupolia rubens *(Heteronemertea). Mitochondrial gene arrangements, structures, and compositions, as well as translation and initiation codons and codon usage patterns, were compared with complete mtDNA sequences of other nemerteans. In addition, we compare gene order among Lophotrochozoa and we use the nucleotide sequences to analyze phylogenetic relationship among the included nemerteans.

## Results and discussion

### Genome organization and structure

**Figure 1:**
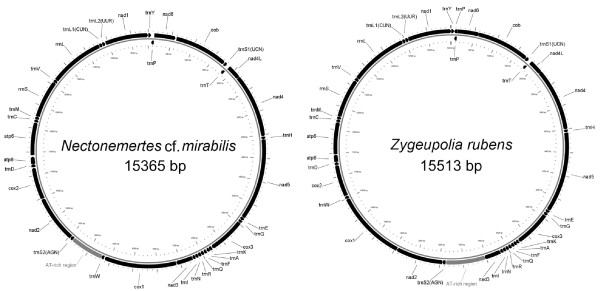
**Circular representation of the mtDNA of *Nectonemertes *cf. *mirabilis *and *Zygeupolia rubens***. Genes on the outer (H) strand are transcribed clockwise; those on the inner (L) strand are transcribed counter-clockwise. Transfer RNA genes are designated by the one-letter amino acid code for the corresponding amino acids; *trnL1, trnL2, trnS1*, and *trnS2 *differentiated on the basis of their codons CUN, UUR, UCN, and AGN, respectively. AT-rich non-coding region is represented in grey. The other small non-coding regions are not marked.

Genome composition and gene arrangement of *Nectonemertes *cf. *mirabilis *and *Zygeupolia rubens *are summarized in Figure 1 and Table 1. The mitochondrial genomes of *N*. cf. *mirabilis *and *Z. rubens *are circular DNA molecules of 15365 bp and 15513 bp, respectively. Lengths of the two nemertean mitochondrial genomes are within the range of previously sequenced nemertean mtDNAs - 14558 bp in *Paranemertes *cf. *peregrina *to 16296 bp in *Cephalothrix hongkongiensis *[6]. Both of the newly sequenced mitochondrial genomes contain 37 genes, including 13 protein-coding genes, two ribosomal RNAs, and 22 transfer RNAs. All genes except *trnP *and *trnT *are encoded on the same strand (Figure 1).

**Table 1:** Location of genes in the mitochondrial genomes of *Nectonemertes *cf. *mirabilis *and *Zygeupolia rubens*

*Nectonemertes *cf. *mirabilis*				*Zygeupolia rubens*			
**Genes**	**From 5'to 3'**	**Size (bp)**	**3'spacer**^**a**^	**Genes**	**From 5'to 3'**	**Size (bp)**	**3'spacer**^**a**^

*trnY*	1-62	62	0	*trnY*	1-64	64	0

*trnP*^b^	125-63	63	2	*trnP*^b^	131-65	67	3

*nad6*	128-583	456	21	*nad6*	135-599	465	-8

*cob*	605-1741	1137	9	*cob*	592-1728	1137	-1

*trnS1 *(UCN)	1751-1811	61	-1	*trnS1*(UCN)	1728-1798	71	-1

*trnT *^b^	1876-1811	66	2	*trnT *^b^	1861-1798	64	2

*nad4L*	1879-2181	303	-7	*nad4L*	1864-2169	306	-7

*nad4*	2175-3536	1362	6	*nad4*	2163-3509	1347	1

*trnH*	3543-3602	60	0	*trnH*	3511-3574	64	2

*nad5*	3603-5348	1746	-1	*nad5*	3577-5308	1732	0

*trnE*	5348-5410	63	1	*trnE*	5309-5372	64	1

*trnG*	5412-5474	63	2	*trnG*	5374-5438	65	2

*cox3*	5477-6256	780	9	*cox3*	5441-6220	780	6

*trnK*	6266-6332	67	-2	*trnK*	6227-6287	61	-1

*trnA*	6331-6393	63	5	*trnA*	6287-6350	64	0

*trnF*	6399-6464	66	1	*trnF*	6351-6415	65	0

*trnQ*	6466-6532	67	0	*trnQ*	6416-6484	69	0

*trnR*	6533-6598	66	1	*trnR*	6485-6550	66	1

*trnN*	6600-6662	63	2	*trnN*	6552-6616	65	0

*trnI*	6665-6730	66	1	*trnI*	6617-6681	65	1

*nad3*	6732-7085	354	5	*nad3*	6683-7039	357	0

*cox1*	7091-8626	1536	12	AT-rich	7040-7877	838	0

*trnW*	8639-8703	65	0	*trnS2*(AGN)	7878-7949	72	0

AT-rich	8704-9405	702	0	*nad2*	7950-8957	1008	3

*trnS2 *(AGN)	9406-9473	68	-1	*cox1*	8961-10493	1533	0

*nad2*	9473-10480	1008	5	*trnW*	10494-10558	65	3

*cox2*	10486-11166	681	14	*cox2*	10562-11246	685	0

*trnD*	11181-11245	65	0	*trnD*	11247-11312	66	0

*atp8*	11246-11402	157	40	*atp8*	11313-11471	159	5

*atp6*	11443-12132	700	5	*atp6*	11477-12169	693	1

*trnC*	12138-12198	61	0	*trnC*	12171-12232	62	0

*trnM*	12199-12263	65	0	*trnM*	12233-12296	64	0

*rrnS*	12264-13068	805	0	*rrnS*	12297-13132	836	0

*trnV*	13069-13130	62	0	*trnV*	13133-13200	68	0

*rrnL*	13131-14308	1178	0	*rrnL*	13201-14448	1248	0

*trnL1*(CUN)	14309-14372	64	1	*trnL1*(CUN)	14449-14515	67	0

*trnL2*(UUR)	14374-14435	62	2	*trnL2*(UUR)	14516-14582	67	0

*nad1*	14438-15361	924	4	*nad1*	14583-15513	931	0

^a^Negative numbers indicate that genes were overlapping

^b^Genes coding in L strand

For both species, protein-coding genes *nad4L *and *nad4 *share an overlap, by seven nucleotides, and *nad6 *overlaps *cob *by eight nucleotides in *Z. rubens *(Figure 1, Table 1). Such overlaps are common to all known mtDNA genomes of nemerteans [6,8], and are found in many metazoan mtDNAs [10].

### Protein-coding genes

Thirteen protein-coding genes (*cox1*-*cox3, nad1*-*nad6, nad4L, cob, atp6*, and *atp8*) were identified. Mitochondrial genomes often use a variety of nonstandard initiation codons [11]. Except for *nad4 *(GTG), *nad5 *(GTG), *atp8 *(GTG) and *atp6 *(GTT) in *N*. cf. *mirabilis*, and *nad1 *(GTG) and *nad2 *(GTG) in *Z. rubens*, the protein-coding genes of both species begin with ATG. The majority of genes in both species contain the full termination codon TAA or TAG, but some end with T (*atp8 *in *N*. cf. *mirabilis*, and *nad5, cox2 *and *nad1 *in *Z. rubens*). Such abbreviated stop codons are common among animal mitochondrial genes. In *Z. rubens*, the incomplete stop codons are immediately followed by the downstream tRNA gene (Figure 1, Table 1), whose secondary structure has been suggested to act as a signal for the cleavage of the polycistronic primary transcript [12,13]. However, there also are direct junctions pairing ten protein-coding genes in *N*. cf. *mirabilis *(*nad6/cob, nad4L*/*nad4, nad3*/*cox1, nad2*/*cox2*, and *atp8*/*atp6*) and eight in *Z. rubens *(*nad6/cob, nad4L*/*nad4, nad2*/*cox1 *and *atp8*/*atp6*) (Figure 1, Table 1). Here, cleavage signals other than secondary structure of a tRNA gene may initiate processing of the polycistronic primary transcript [14]. For two protein-coding genes (*nad6 *and *nad2*) in both nemertean species and *nad3 *in *N*. cf. *mirabilis*, stem-loop structures were inferred to be at the 3' end and abutting the 5' end of the neighboring protein-coding gene, and may signal cleavage of the immature mRNA [15,16].

### Transfer RNA and ribosomal RNA genes

**Figure 2:**
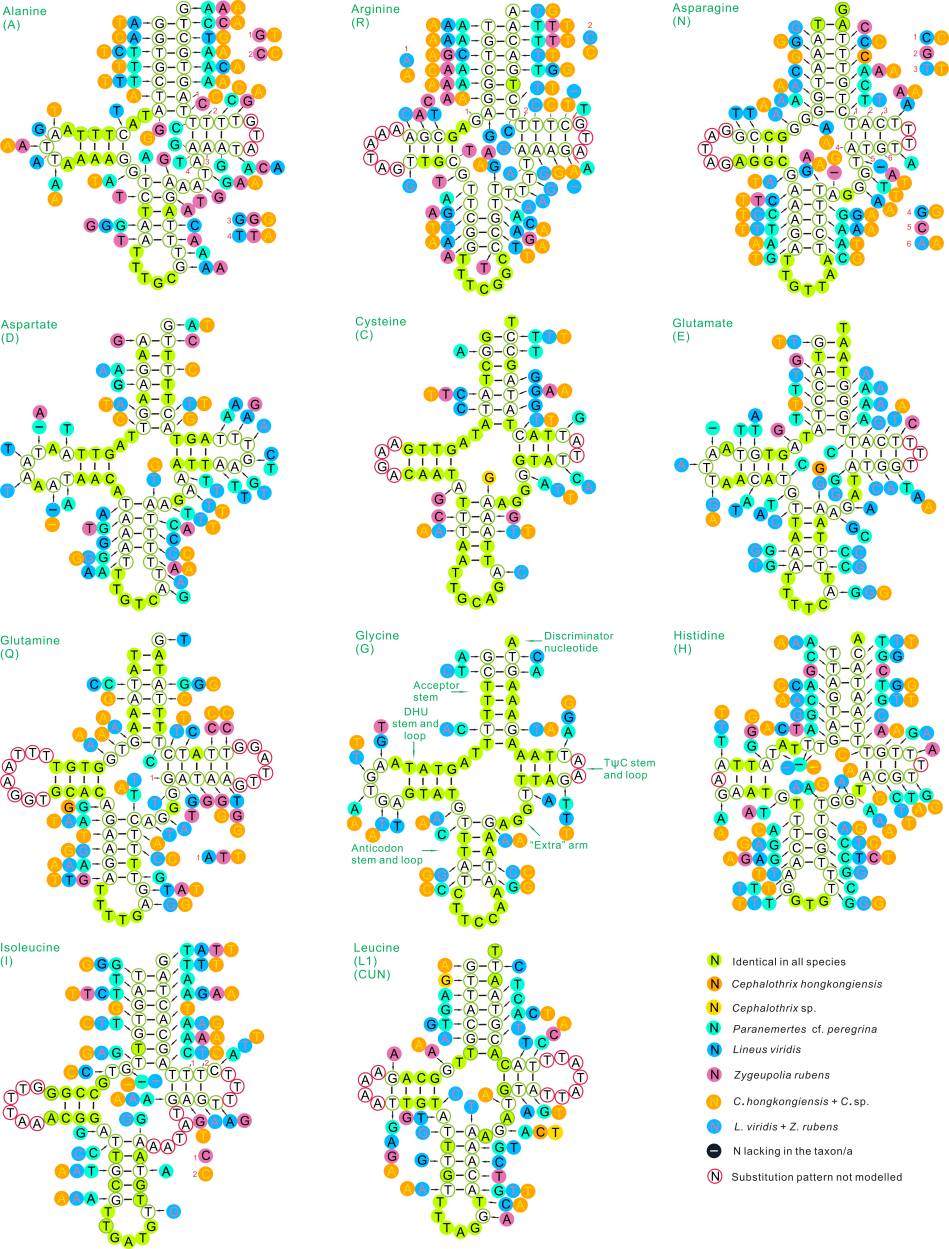
**Secondary structure of tRNA families (*trnA*-*trnL1*) in nemertean mtDNAs**. The nucleotide substitution pattern for each tRNA family was modeled using as reference the structure determined for *Nectonemertes *cf. *mirabilis*.

**Figure 3:**
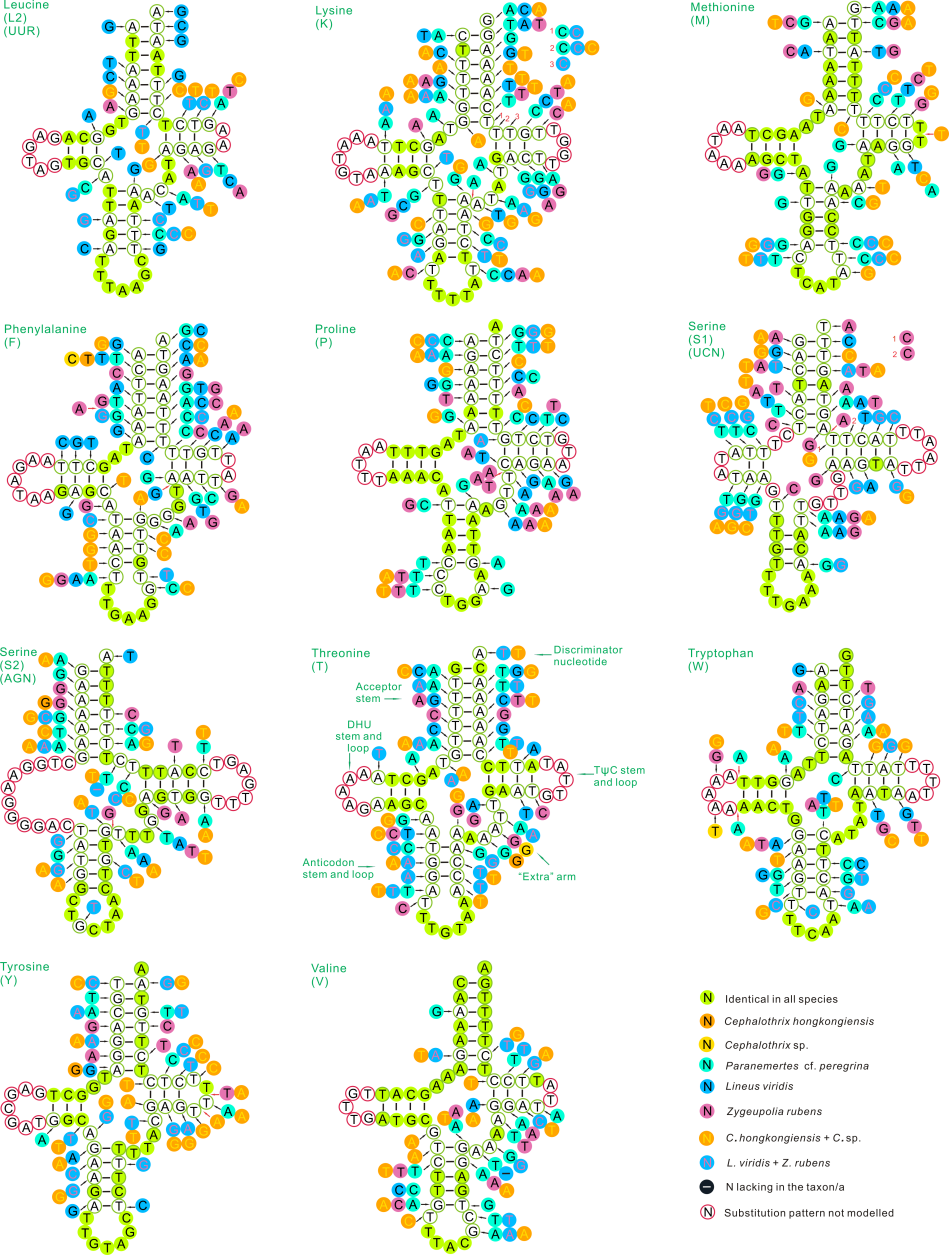
**Secondary structure of tRNA families (*trnL2*-*trnV*) in nemertean mtDNAs**. The nucleotide substitution pattern for each tRNA family was modeled using as reference the structure determined for *Nectonemertes *cf. *mirabilis*.

Both of the mitochondrial genomes encoded 22 tRNA genes found in other nemertean mtDNAs, which is typical of animal mitochondrial genomes [10]. They varied from 60 (*trnH*) to 68 (*trnS2*) nucleotides in *N*. cf. *mirabili*s and 61 (*trnK*) to 72 (*trnS2*) nucleotides in *Z. rubens *(Table 2); most were folded into a typical cloverleaf secondary structure (Figures 2, 3). The postulated tRNA cloverleaf structures generally contained 7 bp in the aminoacyl stem, 2 to 5 bp in the TψC stem, 5 bp in the anticodon stem, and 0 to 4 bp in the dihydrouridine (DHU) stem. Some tRNAs showed DHU-loop replacement (e.g., *trnS1 *of *N*. cf. *mirabili*s), as also found in *L. viridis *and *P*. cf. *peregrina*. In general, the lack of a DHU arm in two serine tRNAs is a common condition in metazoan mtDNAs [17]. The presence of such aberrant tRNA genes in mitochondrial genomes could be due to modification of tRNA secondary structure by replication slippage [18], or selection for mitochondrial genome minimization [19].

**Table 2:** Base composition of the mtDNA in six nemerteans

Species	Total nt	T	C	A	G	A + T	AT skew	GC skew	References
*Cephalothrix hongkongiensis*	16296	47.4	10.2	27.5	14.9	74.9	-0.266	0.187	[6]

*Cephalothrix *sp.	15800	47.9	10.0	27.8	14.3	75.7	-0.266	0.178	[8]

*Paranemertes *cf. *peregrina*	14558	47.5	10.0	22.8	19.7	70.3	-0.351	0.322	[8]

*Nectonemertes *cf. *mirabilis*	15365	48.5	10.5	21.8	19.2	70.3	-0.380	0.293	Present study

*Lineus viridis*	15388	44.4	11.9	21.3	22.4	65.7	-0.352	0.306	[7]

*Zygeupolia rubens*	15513	45.0	9.8	21.0	24.2	66.0	-0.364	0.424	Present study

The mtDNAs of nemerteans investigated to date all have 20 tRNAs on the L strand and 2 tRNAs on the H strand ([6-9]). Secondary structures of nemertean tRNAs are presented and compared in Figures 2 and 3 (pattern follows [20]). Table 3 presents the tRNA lengths and the percent of identical nucleotides (%INUC) for the six nemerteans.

**Table 3:** Summary of multiple alignments of tRNA genes in nemertean mtDNAs

ALN	amino acid	alignment length	identical positions	%INUC
*trnA*	Alanine	72	21	29.17

*trnC*	Cysteine	66	39	59.09

*trnD*	Aspartate	66	26	39.39

*trnE*	Glutamate	65	27	41.54

*trnF*	Phenylalanine	68	22	32.35

*trnG*	Glycine	67	39	58.21

*trnH*	Histidine	67	25	37.31

*trnI*	Isoleucine	72	28	38.89

*trnK*	Lysine	73	23	31.51

*trnL1*	Leucine (CUN)	69	21	30.43

*trnL2*	Leucine (UUR)	68	28	41.18

*trnM*	Methionine	66	38	57.58

*trnN*	Asparagine	69	20	28.99

*trnP^a^*	Proline	67	24	35.82

*trnQ*	Glutamine	70	28	40.00

*trnR*	Arginine	67	16	23.88

*trnS1*	Serine (UCN)	71	23	32.39

*trnS2*	Serine (AGN)	73	30	41.10

*trnT^a^*	Threonine	71	23	32.39

*trnV*	Valine	69	33	47.83

*trnW*	Tryptophan	70	27	38.57

*trnY*	Tyrosine	68	32	47.06

ALN, alignment name; %INUC, percent of identical nucleotides

^a^genes on the L strand

Nucleotide conservation was strongest on the H strand, with *trnC, trnG *and *trnM*, having the highest levels of nucleotide conservation (%INUC > 50), followed by *TrnE, trnL2, trnQ, trnS2, trnV *and *trnY *at 40 ≤ %INUC ≤ 50 (Figure 2). The ten remaining tRNAs had %INUC values between 30 and 40; eight - *trnD, trnF, trnH, trnI, trnK, trnL1, trnS1 *and *trnW *- are located on the H strand, while two - *trnP *and *trnT *- are on the L strand. H-strand genes *trnA, trnN *and *trnR *had %INUC values ≤30.

Conservation was positively H strand-biased, but no other pattern could be identified with respect to location of tRNAs along the genome. Two of the three most conserved tRNAs, *trnC *and *trnM*, are adjoining, while the third, *trnG*, adjoins the moderately conserved *trnE *and is relatively close to the three least conserved genes, *trnA, trnN *and *trnR *(Figure 1, Table 1). As observed by others (e.g., [20]), there was no self-evident link between abundance of codon families and the level of tRNA conservation, with the most abundant codon families (Leu2, Ile and Phe) not having the highest %INUCs (see below).

A few mismatched nucleotide pairs (e.g., G-A, A-A, T-C, T-T) were found in the acceptor and/or the discriminator arms, without regard to the overall level of conservation of the tRNAs. As recently pointed out by Negrisolo et al. [20] for arthropods, metazoan mtDNAs commonly have such mismatches. It has been suggested that these may be corrected via RNA-editing mechanisms (e.g., [17]) or they may represent unusual pairings [21].

Among the most conserved tRNAs in nemerteans, as in insects (e.g., [20]), nucleotide substitutions are mostly confined to TΨC and DHU loops and extra arms (Figures 2, 3), with 2-7 fully compensatory base changes (cbc; e.g., G-C vs. A-T) or hemi-cbcs (e.g., T-A vs. T-G) on acceptor and anticodon stems (see [20,22]). As in insects [20], the number of cbcs and hemi-cbcs increased in stems as overall variation increased, especially in the TΨC stem.

As found in insects, cbcs and hemi-cbcs characterized either single species or taxa at a higher taxonomic rank. An example of the first type is the A-T pair found in the *trnC *acceptor arm of *P*. cf. *peregrina*, which was mirrored by G-C in all other nemerteans (Figure 2). Few substitutions were present among *C. hongkongiensis *and *Cephalothrix *sp. (Figures 2, 3). An example of a full cbc characterizing a unique family is the A-T pair found in the acceptor stem of *trnL1s *of family Lineidae (*L. viridis *and *Z. rubens*), while other taxa exhibited the G-C pair (Figure 2). Similarly, a full cbc in the anticodon stem of *trnG *of two hoplonemerteans characterizes another high-taxonomic rank (Figure 2). Figures 2 and 3 depict several more examples. This points to the potential phylogenetic value of tRNA sequences, as demonstrated for other animal groups (e.g., [20,23]), especially when secondary structures are taken into account [20]. While encouraging, clearly we need substantially more nemertean mitochondrial genomes to test this assertion for nemerteans.

The anticodon usage of *N*. cf. *mirabili*s and *Z. rubens *was congruent with the corresponding tRNA genes of other nemerteans, with one exception. The anticodon of the *trnS2 *(AGN) gene in *N*. cf. *mirabili*s, *P*. cf. *peregrina *and three *Cephalothrix *species is GCT, but it is TCT in *L. viridis *and *Z. rubens*. Cameron et al. [24] found that anticodon changes in *trnS2 *(AGN) (GCT→TCT) must have occurred in the common ancestor of the insect clade Ischnocera, which was consistent with its phylogeny of lice. Similarly, this may constitute a kind of "rare genomic change" [25] in nemerteans and be a synapomorphy of Lineidae.

As in all other metazoan mtDNAs sequenced to date, *N*. cf. *mirabili*s and *Z. rubens *mtDNAs contain genes for both small and large ribosomal subunit RNAs (*rrnS *and *rrnL*). Both genes are encoded by the same strand and are separated by *trnV*, as in many other metazoans. For *N*. cf. *mirabili*s and *Z. rubens*, respectively, the lengths of *rrnL*/*rrnS *are 1178/805 bp and 1248/836 bp, and the A + T contents are 75.5/72.4% and 70.9/70.5%.

### Base composition and codon usage

The mtDNA of many invertebrates is characterized by a composition bias showing high values of A% and T% over G% and C%. The overall A + T content of *N*. cf. *mirabili*s and *Z. rubens *(70.3% and 66.0%, respectively) is consistent with those observed in other nemertean mitochondrial genomes. Though sample size for nemerteans is small, the A + T values appear to be linked in less (e.g., genus - e.g., *Cephalothrix *sp./*C. hongkongiensis*), as well as in more inclusive taxa (e.g., order - e.g., *P*. cf. *peregrina*/*N*. cf. *mirabili*s; *L. viridis/Z. rubens*) (Table 2). This might indicate a phylogenetic signal in nemerteans.

**Figure 4:**
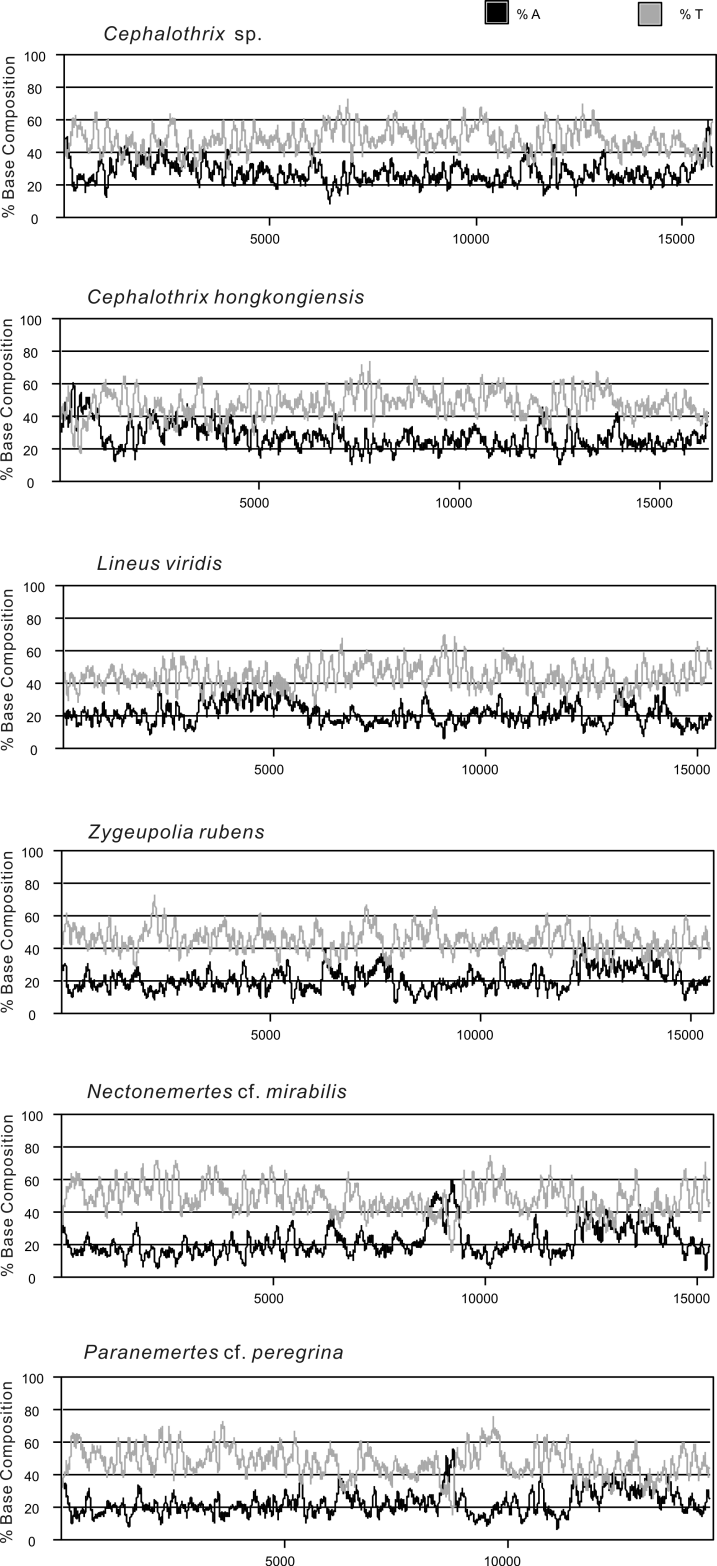
**Graphical representation of the percentage of A (black) and T (gray) across the whole mtDNA segment of six nemertean species (Accelrys)**. Y-axis values represent nucleotide %, calculated with a 100-bp sliding window using the program MacVector^® ^7.2.3; x-axis values represent the nucleotide positions corresponding to the linearized genome.

Another feature of metazoan mtDNAs is asymmetry in nucleotide composition between the two strands, with one being rich in A and C, and the other being rich in T and G [26]. This asymmetry also is evident in the two nemertean mtDNA genomes here, with the genes encoded on the coding strand showing a strong bias toward T over A and toward G over C, as seen in the four other nemerteans, which have similar skewnesses (Table 2; Figure 4). This situation is common for mitochondrial genomes [26] and may be due to the presence of asymmetric patterns of mutational changes between strands [27,28], and has been related with nucleotide deamination of DNA while transiently single-stranded during replication (this is not without controversy [29]) and/or transcription [30]. The relative importance of the two contributing processes (i.e., transcription vs. replication) remains to be assessed.

**Figure 5:**
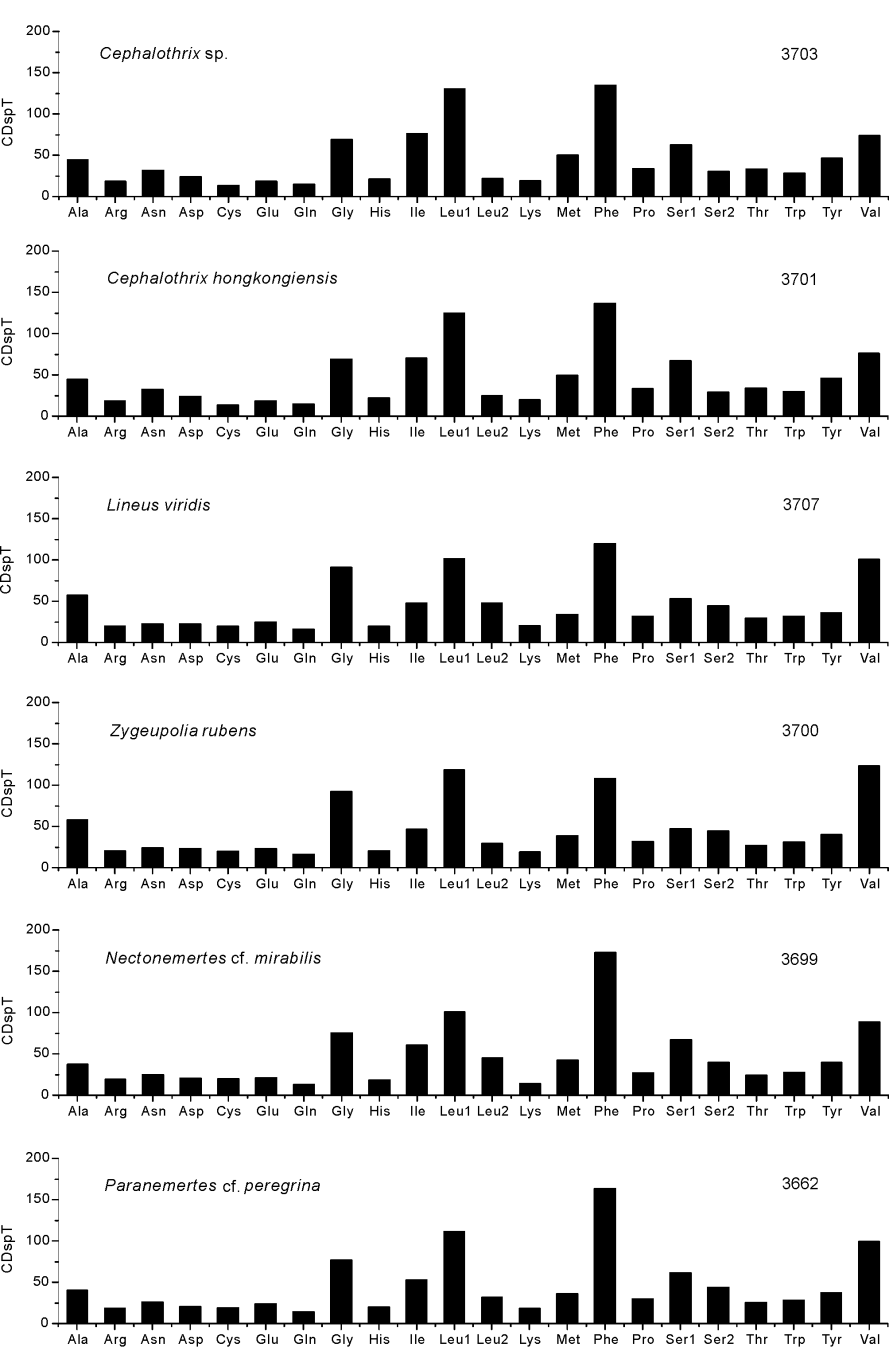
**Codon distribution in nemertean mtDNAs**. CDspT, number of codons per thousands codons. Numbers to the right refer to the total number of codons.

**Figure 6:**
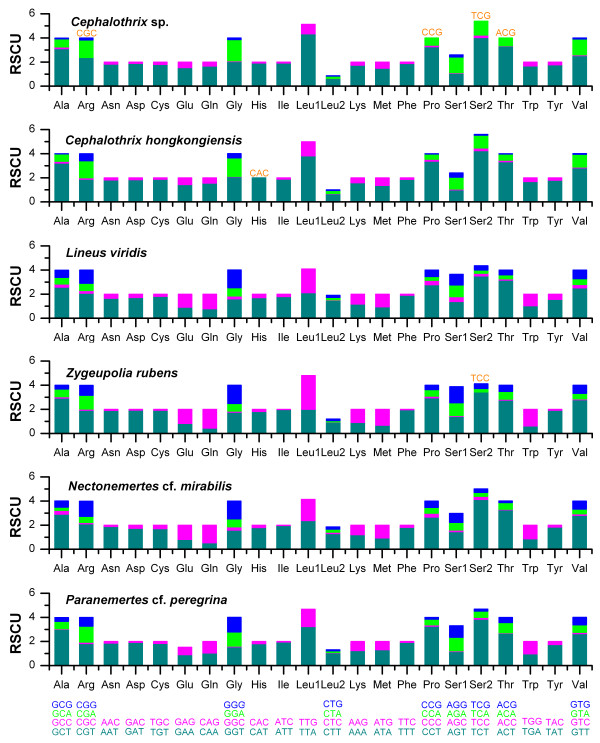
**Relative Synonymous Codon Usage (RSCU) in nemertean mtDNAs**. Codon families are provided on the x-axis, codons not present in the genome are orange colored.

We follow the pattern of [2] for displaying codon family abundance and relative synonymous codon usage (RSCU) for available nemertean protein-coding genes (Figures 5 and 6). To avoid bias due to incomplete stop codons, all stop codons are excluded from the analysis. The six nemertean mtDNAs use similar total numbers of non-stop codons (CDs), ranging from 3662 in *P*. cf. *peregrina *to 3707 in *L. viridis*. The codon families reveal a consistent pattern among the six nemertean species: the families with at least 50 CDs per thousand CDs (Leu1, Ile, Phe, Gly, Val) encompass an average 48.78% ± 1.33% of all CDs (Figure 5), with CDs rich in A + T favored over synonymous CDs of lower A + T content (Figure 6). For instance, the TTA codon accounts for a large majority of CDs in the Leu1 family. Whereas representation of the Leu1 (average = 77.3 ± 7.3%) and Leu2 (average = 22.7 ± 7.3%) codon families in nemertean protein-coding genes differs greatly, that of Ser1 (average = 60.8 ± 7.3%) and Ser2 (average = 39.2 ± 7.3%) is less extreme.

The invertebrate mitochondrial genome codes for 62 amino-acid codons [10]. As pointed out for Lepidoptera [2], the total number of codons used seems to be linked to the A + T content, which is the case among the six nemertean genomes analyzed. Thus, *Cephalothrix *sp. mtDNA has the highest (A + T)% content (see Table 2) and uses only 58 codons, never using the four codons rich in G + C (TCG, CGC, ACG, CGC) (Figure 6). *Lineus viridis *mtDNA uses all 62 codons and has the lowest A + T% among known nemertean genomes.

The abundance of the four amino acid residues - Leu, Ile, Phe and Ser - is typical for invertebrate membrane proteins [2,31], and they account here for more than 46.70% (average A + T = 50.14 ± 2.70%) of residues comprising the 13 mitochondrial proteins. The Leu and Ile amino acids share hydrophobic lateral chains.

Two- and four-fold degenerate codon usage was similarly biased, with A/T favored over G/C in the third position (Figure 6) and in agreement with the AT-bias of protein-coding genes. Since the nemertean mitochondrial genome is AT-rich (Table 2), it can be expected that codons ending in A or T will predominate. From the overall RSCU values, it could be assumed that compositional constraints are the factor in shaping variation in codon usage among the genes in these mitochondrial genomes.

### Non-coding regions

Metazoan mtDNAs usually have lengthy non-coding regions varying in size from ~100 bp to > 20 kbp [32,33]. The mtDNAs of *N*. cf. *mirabili*s and *Z. rubens *contain a large number of unassigned nucleotides. There are 23 non-coding regions, with up to 855 nts, found throughout the *N*. cf. *mirabili*s mitochondrial genome. The AT-rich region located between the *nad3 *and *trnS2 *genes accounts for 838 nts and its AT content is 81.5%, which is higher than the remainder of the genome. *Zygeupolia rubens *has up to 879 non-coding nts distributed in 15 regions. The AT-rich region located between *trnW *and *trnS2 *genes is 702 nts and has an AT content of 74.9%, which also is higher than the remainder of the genome.

**Figure 7:**
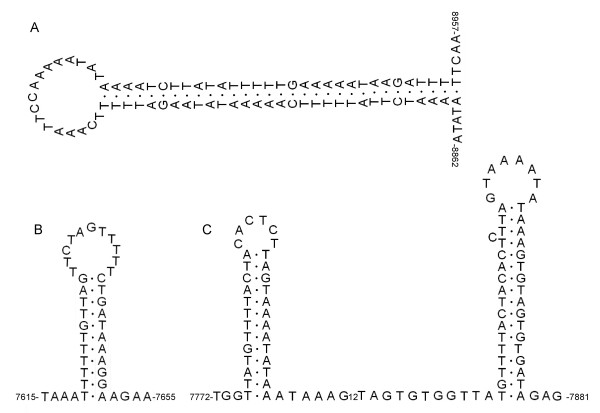
**Secondary structures predicted for the non-coding regions in the mitochondrial genome of two nemerteans**. (A) *Nectonemertes *cf. *mirabilis*, AT-rich non-coding region between genes *trnW *and *trnS2*; (B, C) *Zygeupolia rubens*, AT-rich non-coding region between genes *nad3 *and *trnS2*.

In most metazoan mtDNAs, the largest non-coding region is thought to contain signals for replication and transcription, and is thus referred to as the control region [11]. The non-coding region has an increased AT composition, a characteristic typically used to identify origins of replication [10]. As in mtDNA genomes of other nemerteans, the AT-rich regions of *N*. cf. *mirabili*s and *Z. rubens *mtDNAs have the potential to form secondary structures such as stems and loops (Figure 7), which are thought to play an important role in the early stages of the replication and transcription process [34,35]. Additionally, the AT-rich region in mtDNA of *N*. cf. *mirabili*s contains the tandemly repeated sequences (AAAAATATAAGATTTTTCAAATTCCAAAAATATAAAAT)_3_, (TTTTG)_10_, (TTTTTC)_7_, and several (A)_n _and (T)_n _homopolymer tracts. In mtDNAs of *Z. rubens*, we found the tandemly repeated sequences (GGGGGGGGGGGTAGTGTGGTTATGTTTTACTACACTCTTAGTAAAATATAAA)_2_, (TTTTTTG)_10_, and (TTTTTTTTA)_6_. Similar tandem repeat units within the largest non-coding regions also were found in the nemerteans *Cephalothrix *sp. [8], and *C. hongkongiensis *[6]. Tandem repeats are common within the control region of animal mtDNAs [34] and might be associated with regulatory mechanisms and recombination hot spots, and they might be the result of replication slippage events [36]. The high AT content and the predicted secondary structures of the AT-rich non-coding region of the *N*. cf. *mirabili*s and *Z. rubens *mtDNAs suggest that this region most likely contains the control region, though the control region in invertebrates, unlike that of vertebrates, is not well characterized and lacks discrete and conserved sequence blocks used in identification [37]. The nemertean mtDNA sequences examined here had multiple non-coding regions throughout their genomes. However, the location of the largest non-coding region is not conserved, and there is no obvious conservation of size (e.g., [6,8]), nucleotide identities or potential secondary structures for the nemertean non-coding regions.

### Gene order comparison

Gene arrangements of the animal mitochondrial genome usually remain stable over long periods of evolutionary time, especially for protein-coding genes [10]. With some exceptions, mitochondrial gene order is relatively stable within major groups, and more variable between them [14]. This is the case for available nemertean mtDNA genomes, with mitochondrial genes transcribed from the same strand except for *trnP *and *trnT*. Among the three species of *Cephalothrix *(*C. hongkongiensis, C*. sp. and *C. rufifrons*), the gene order is identical for two but that of *C. rufifrons *differs from them. The two hoplonemertean species (*P*. cf. *peregrina, N*. cf. *mirabili*s) are identical to each other in gene order, as is the case for the two heteronemerteans (*Z. rubens, L. viridis*). The hoplo- and the heteronemertean species differ only by a translocation of the gene block S2/*nad2 *but they differ significantly from the three *Cephalothrix *species in the positions of *atp8, nad6, nad2 *and several tRNAs. The highest number of common intervals (1124) is between hoplo- and heteronemerteans, as indicated by results from CREx [38].

**Figure 8:**
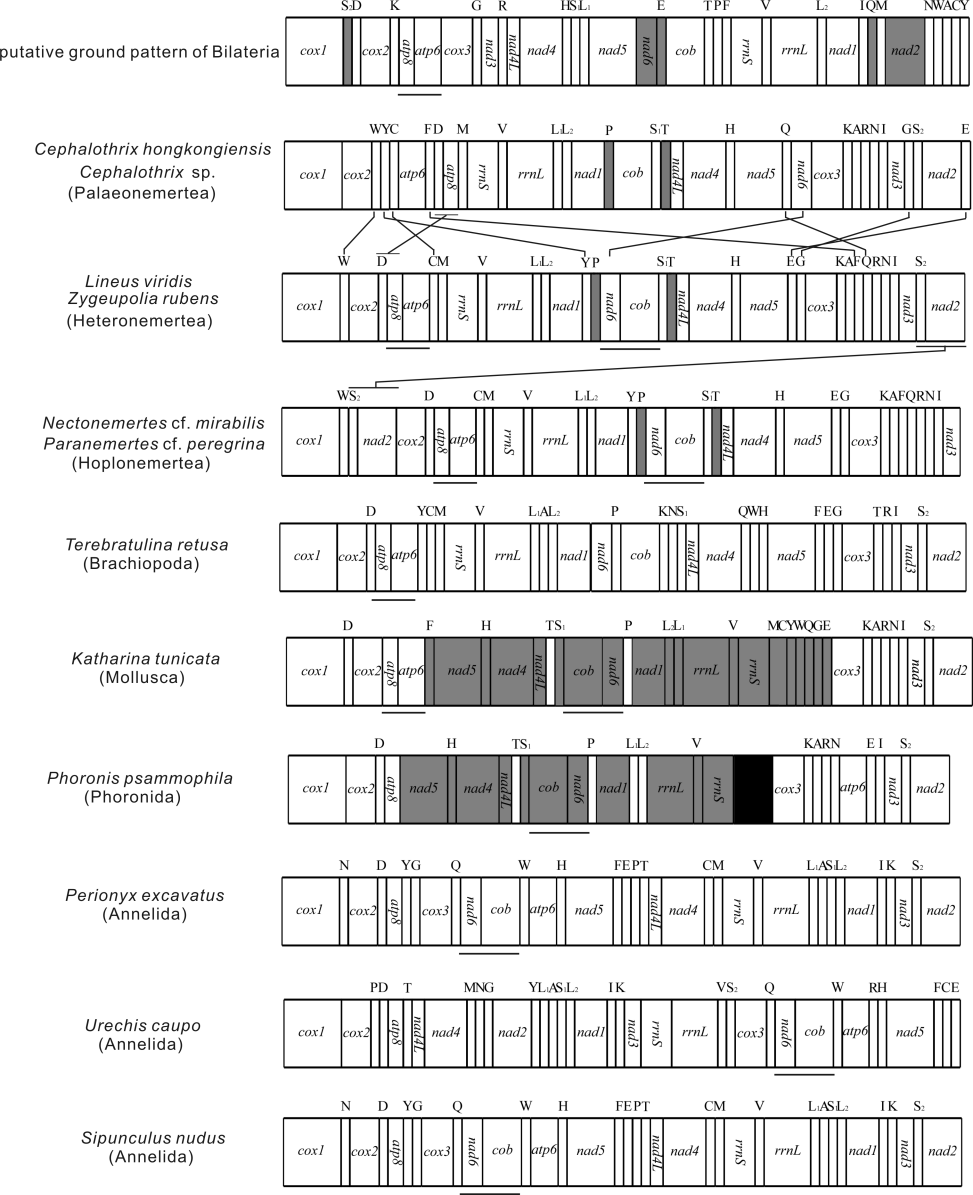
**Mitochondrial gene order (all 37 genes) of Nemertea and selected lophotrochozoan species and the putative bilaterian ground pattern (according to **[39]**)**. Gene segments are not drawn to scale. All genes are transcribed from left to right except those in gray, which are transcribed from right to left. Unsequenced regions are in black. The adjacencies nad6/cob and atp8/atp6 are underlined. Previous gene orders from the following references: *Cephalothrix *[6,8], *Lineus *[7], *Paranemertes *[8], *Terebratulina *[40], *Katharina *[14], *Phoronis *[41], *Perionyx *[42], *Urechis *[43], *Sipunculus *[44].

We use two different gene sets, "all genes" and "non-tRNA genes" to compare the mt gene orders of nemerteans to the proposed ground pattern of Bilateria [39] and to mitochondrial gene orders of various lophotrochozoans: *Terebratulina retusa *(Brachiopoda) [40], *Katharina tunicata *(Mollusca)[14], *Phoronis psammophila *(Phoronida) [41], *Perionyx excavatus *(Annelida) [42], *Urechis caupo *(Annelida) [43] and *Sipunculus nudus *(Annelida)[44]. For the "all genes" set, all nemerteans share the adjacency *nad4L/nad4 *with the ground pattern of Bilateria and with the selected species (Figure 8). Nemerteans share the adjacencies *rrnS*/V/*rrnL *with Bilateria and the other species except *U. caupo*. The adjacency H/*nad5 *is shared by nemerteans and the selected species. Based on both gene sets, the hetero- and hoplonemerteans share the adjacency *nad6/cob *with *K. tunicata *[14], *P. psammophila *[41], *P. excavatus *[42], *U. caupo *[43], and *S. nudus *[44] and they share the adjacency *atp8/atp6 *with *T. retusa, K. tunicata *and the putative ground pattern of Bilateria (Figure 8; Additional file 1: Figure S1). These adjacencies may be a common plesiomorphic feature of Lophotrochozoa, such as Mollusca, Brachiopoda, and also Arthropoda mitochondrial genomes (e.g., [10]; [44]). However, neither of the latter two adjacencies is present in two *Cephalothrix *species, nor in the bryozoan *Flustrellidra hispida *[45].

In addition to visual comparison of genome maps, we analyzed gene order data with CREx [38], determining the number of common intervals. As shown in Table 4, the nemerteans share the highest number of common intervals (154, 184, 212) with *K. tunicata *and with *P. psammophila *(but this is a partial mitochondrial genome), while the lowest number was obtained in comparison with *U. caupo *(28, 18, 18). Although not significant, nemerteans and *T. retusa, K. tunicata*, and *P. excavatus *yield the highest numbers (18-20) in comparison with the putative bilaterian ground pattern.

**Table 4:** Pairwise common interval distance matrix of mitochondrial gene orders of nemerteans, the putative bilaterian ground pattern and six other lophotrochozoans *

Common interval	B	P	H	H	Tr	Kt	Uc	Sn	Pe	Pp
Bilaterian ground pattern (B)	204\**1326**	**18**	**20**	**20**	**18**	**20**	**12**	**14**	**20**	**12**

Palaeonemertean (P)	*44*	204\**1326**	**108**	**112**	**40**	**154**	**28**	**42**	**38**	**142**

Heteronemertean (H)	*52*	*86*	204\**1326**	**1124**	**68**	**184**	**18**	**64**	**56**	**230**

Hoplonemertean (H)	*44*	*72*	*178*	204\**1326**	**84**	**212**	**18**	**68**	**66**	**254**

*Terebratulina retusa *(Tr)	*52*	*86*	*204*	*178*	204\**1326**	**128**	**20**	**74**	**82**	**110**

*Katharina tunicata *(Kt)	*48*	*56*	*106*	*94*	*106*	204\**1326**	**20**	**62**	**64**	**266**

*Urechis caupo *(Uc)	*16*	*8*	*14*	*8*	*14*	*34*	204\**1326**	**54**	**144**	**22**

*Sipunculus nudus *(Sn)	*34*	*12*	*22*	*16*	*22*	*26*	*26*	204\**1326**	**158**	**38**

*Perionyx excavatus *(Pe)^a^	*28*	*24*	*40*	*32*	*40*	*48*	*44*	*60*	204\**1254**	**44**

*Phoronis psammophila *(Pp)^b^	*40*	*48*	*84*	*76*	*84*	*98*	*22*	*24*	*38*	204\**864**

*bold numbers represent pairwise common interval distances between mitochondrial gene orders (37 genes in total), while italic numbers represent pairwise common interval distances between mt gene orders without tRNAs (15 genes in total)

^a^lacks *trnR*

^b^lacks several tRNAs

Figure 8 shows tRNA genes change relative position much faster than the protein-coding and rRNA genes, as reported from previous studies (e.g., [46,47]).

Excluding tRNAs, the gene order of heteronemerteans is identical to that of *T. retusa *[40] and some gastropods, e.g., *Conus textile *[48], *Ilyanassa obsoleta *[49], *Thais clavigera *[37] and *Lophiotoma cerithiformis *[50]. Other molluscs, like the polyplacophoran *K. tunicata *[14], the gastropod *Haliotis rubra *[51] and the cephalopod *Octopus vulgaris *[52] show a similar gene order, but are distinguished by a large inversion of about half the mt genome (Additional file 1: Figure S1). Without tRNAs, heteronemerteans and *T. retusa*, which has the same gene order, share the greatest number of possible common intervals (204) (Table 4), and both share the greatest number (52) with the putative bilaterian ground pattern.

We also determined breakpoints and reversal distances between these taxa with the two gene sets (Additional files 2, 3: Tables S1, S2). For "all genes", hetero- and hoplonemerteans share the same breakpoint distance (31) and the same reversal distance (28) (whereas palaeonemerteans are 32 and 31, respectively) with respect to the putative bilaterian ground pattern. Heteronemerteans have the lowest values among the nemerteans when tRNAs are excluded from the analysis. These comparisons with the putative bilaterian ground pattern and with other lophotrochozoans gene orders (especially when excluding tRNAs), suggest that the heteronemertean gene order is likely to be closest to the ancestral mitochondrial gene order of Nemertea. This is in agreement with a previous study [7].

### Phylogenetic analysis

**Figure 9:**
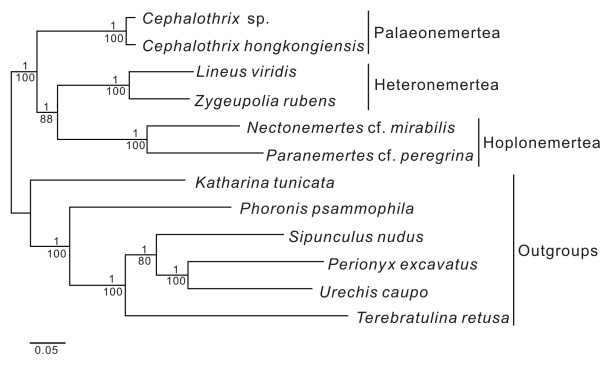
**Best tree from the Maximum Likelihood analysis with 5921 nt (first and second codon positions) of protein-coding genes**. Node support is indicated above (Bayesian posterior probabilities) and below (maximum likelihood bootstrap values) each branch. A Bayesian analysis resulted in the same species topology.

We performed a phylogenetic analysis based on nucleotide sequences of protein-coding genes to better understand relationships within the Nemertea. The phylogenetic tree in Figure 9, reconstructed by maximum likelihood and Bayesian analyses, indicates that similar gene arrangements reflect close phylogenetic affinity. This supports previous hypotheses that Hoplonemertea and Heteronemertea are sister taxa (e.g., [53-55]). However, a phylogenetic analysis based on amino acid sequences (data not shown) suggests Hoplonemertea as sister group to Palaeonemertea. This contradicts many but not all previous analyses (e.g., [55]). We consider it unsupported by our data, given the low Bayesian posterior probability (0.61) for this clade. This suggests, however, that amino acid sequence data deserve continued attention in future analyses with new, additional data.

## Conclusion

To date, complete or nearly complete mtDNA sequences have been determined for seven nemerteans, a very small sampling compared to those available for vertebrates or arthropods. The two new mtDNA genomes, for *Nectonemertes *cf. *mirabilis *and *Zygeupolia rubens*, share substantial similarity with those of other nemertean mitochondrial genomes, and gene content and A + T richness is similar to those common for animal mtDNAs.

There is strong skew in the distribution of nucleotides between the two strands.

The evolution of nemertean tRNAs seems to have been variable both in terms of sequence conservation and nucleotide substitution processes. The presence of full and hemi-cbcs characterizing taxa at different taxonomic levels may indicate the potential phylogenetic value of tRNA sequences.

Nemertean mtDNAs are punctuated by non-coding portions highly variable in size. Among them, the AT-rich non-coding region, which appears to be a fast-evolving genomic region characterized by short to medium-size repeated motifs/AT-rich patterns, may be associated with the initiation of replication or transcription.

Phylogenetic analysis supports the close phylogenetic affinities in nemerteans one might infer from similarities in gene arrangements, with Hetero- and Hoplonemerteans as sister-clades. Gene order analysis suggests that the heteronemertean pattern most closely resembles the likely ancestral condition among nemerteans, which is counterintuitive in light of the phylogenetic analysis. Confidence that we understand evolution of the nemertean mitochondrial genome is likely to require investigating many more nemertean mtDNAs, especially a full representation of palaeonemertean diversity.

## Methods

### DNA extraction, PCR and sequencing

Specimens were collected off Point Conception, California (*Nectonemertes *cf. *mirabilis*) and at Fort Pierce, Florida (*Zygeupolia rubens*), USA. We use the "cf." qualifier to confer reasonable caution that the Pacific worm traditionally designated *N. mirabilis *(see [56]) is conspecific with its presumed cognate originally described from the North Atlantic Ocean. Samples were frozen in liquid nitrogen and preserved in RNAlater. Total DNA was extracted from a single individual specimen using the DNeasy Tissue Kit following the manufacturer's protocol (Qiagen, Valencia, CA, USA). PCR primers used for amplification are listed in Table 5. Preliminary nemertean-specific primers (N12SF, N16SR, NCOX2R) were designed based on sequence alignment of four mitochondrial genome sequences (*Cephalothrix hongkongiensis, Cephalothrix*. sp., *Lineus viridis*, and *Paranemertes *cf. *peregrina*) retrieved from Genbank. For both species, the partial regions *rrnS-rrnL *and *rrnL*-*cob *were amplified first. For *N*. cf. *mirabilis*, partial fragments of *cox1 *and *cox3 *genes were amplified using universal PCR primers LCO-2198/HCO-1490, cox3F/cox3R ([59]; [9]). These sequences were used to design specific primers to amplify the remaining mitochondrial genome fragments (*cob-cox3, cox3-cox1 *and *cox1-rrnS*). For *Z. rubens*, the fragment of *cox1-cox2 *was amplified using the universal primer LCO-2198 [59] combined with the specific primer NCOX2R. Based on sequences obtained, specific primers were designed to amplify the regions *cox2-rrnS, cob-cox3 *and *cox3-cox1*. Conventional PCR and long PCR, cloning, and sequencing were performed as described in Chen et al. [6,8].

**Table 5:** PCR primers used to amplify the mitochondrial genomes of *Nectonemertes *cf. *mirabilis*

Primer name	Sequence (5' → 3')	References
Universal		

*rrnS-rrnL*		

N12SF	TGTGCCAGCTTCCGCGGTTATAC	Present study

N16SR	ACGCTGTTATCCCTATGGTA	Present study

*rrnL-cob*		

16SarL	CGCCTGTTTATCAAAAACAT	[57]

CytbR	GCRTAWGCRAAWARRARTAYCAYTCWGG	[58]

*Nectonemertes *cf. *mirabilis*		

*cox1*		

LCO-1490	GGTCAACAAATCATAAAGATATTGG	[59]

HCO-2198	TAAACTTCAGGGTGACCAAAAAATCA	[59]

*cox3*		

cox3F	TGCGWTGAGGWATAATTTTATTTATT	[8]

cox3R	ACCAAGCAGCTGCTTCAAAACCAAA	[8]

*cob-cox3*		

Nm cobF	TCGGTGGATAATGCTACTTTG	Present study

Nm COX3R	ACCAGAAGCCAACAATACAGC	Present study

*cox3-cox1*		

Nm COX3F	TGTTGGCTTCTGGTGTTAGTG	Present study

Nm COX1R	GAGCCTCTTTCAACAACAGCA	Present study

*cox1-rrnS*		

NmCOX1F	AATCTGGTCTGGGTTGGTTGGCACTGCGTTA	Present study

Nm12SR	GACTCCCCTGAAAGGACATAAAACACCG	Present study

*Zygeupolia rubens*		

*cob-cox3*		

Zrcob F	CTTTGGGTTTGTTGCTGTTG	Present study

ZrCOX3R	GTTGAACCATAAATCCCATC	Present study

*cox3-cox1*		

cox3F	TGCGWTGAGGWATAATTTTATTTATT	[8]

ZrCOX1R	GAGCCTCTTTCAACAACAGCA	Present study

*cox1-cox2*		

LCO-1490	GGTCAACAAATCATAAAGATATTGG	[59]

NCOX2R	AAAGAATGATTWGCWCCAC	Present study

*cox2-rrnS*		

ZrCOX2F	TTTGGCTTTACCTTCTTTGC	Present study

Zr12SR	AAATAAGACACCGCCAAGT	Present study

### Sequence assemblage and annotation

All sequences were checked and assembled by visual inspection using the program SeqMan (DNA star, Madison, WI, USA). Protein-coding genes and ribosomal RNA genes were identified by their similarity to previously reported mitochondrial genomes of *Cephalothrix hongkongiensis *(GenBank #NC_012821), *C. rufifrons *(EF140788), *Cephalothrix *sp. (NC_014869), *Lineus viridis *(NC_012889), and *Paranemertes *cf. *peregrina *(NC_014865). Most tRNAs were identified by using invertebrate mitochondrial codon predictors with tRNAscan-SE 1.21 [60]. The remaining tRNA genes were found by inspecting sequences for tRNA-like secondary structures and anticodons. Multiple alignments of tRNA genes were produced, and the percent of identical nucleotides (%INUC) was calculated for six nemertean tRNA sequences. Secondary structures within the non-coding fragments were visualized by using RnaViz 2.0 [61], and the mitochondrial genome was visualized using CGView [62].

### Genomics analysis

Nucleotide composition and Relative Synonymous Codon Usage (RSCU) values were analyzed with MEGA 4.0 [63]. AT- and GC-skew were determined by using the formulation of [26].

### Gene order comparisons

Gene orders were compared between all available nemerteans (see above), the putative bilaterian ground pattern [39], *Terebratulina retusa *[40], *Katharina tunicata *[14], *Phoronis psammophila *[41], *Perionyx excavatus *[42], *Urechis caupo *[43] and *Sipunculus nudus *[44].

The gene orders were compared with two different gene sets: "all genes" included all 37 mitochondrial genes, whereas "non-tRNA genes" included only the two ribosomal genes and the 13 protein-coding genes.

The differences between gene orders were analysed using common intervals [38], breakpoints [64] and reversal distances [65] implemented in the CREx tool [38].

### Phylogenetic analysis

The currently available near-complete and complete mitochondrial nemertean genome data (*Cephalothrix *sp., *C. hongkongiensis, L. viridis*, and *P*. cf. *peregrina*, but not the partial genome sequence of *C. rufifrons*) were combined with sequences from this study for phylogenomic analyses. The nucleic acids for all 12 protein-coding genes (except *atp8*, which is shortest and least conserved between the taxa) were aligned using Clustal X [66] with the default settings. Ambiguously aligned portions of both alignments were excluded using Gblocks version 0.91b [67] with default block parameters. We also excluded third codon positions because of the fast substitution rate. The total number of nucleotides used for the phylogenetic analysis was 5921.

Based on previous studies of metazoan relationships (e.g., [68-73]), the following six species were selected as outgroups: a mollusc (*Katharina tunicata*), a brachiopod (*Terebratalia retusa*), a phoronid (*Phoronis psammophila*), and three annelids (*Perionyx excavatus, Sipunculus nudus *and *Urechis caupo*).

Phylogenetic trees were estimated under maximum likelihood (ML) and Bayesian inference (BI). ML analysis on the combined nucleotide dataset alignments was performed in RAxML 7.2.7 [74,75] available on the CIPRES web portal [76] with the GTRGAMMA substitution model. Support was estimated by performing 1000 bootstrap replicates. BI analysis was performed with MrBayes version 3.0b4 [77,78], using GTR + I + G model, a best-fit model selected by MrModeltest v2.2 [79] following the Akaike information criterion (AIC). The Monte Carlo Markov chain (MCMC) length was 1,000,000 generations and sampled every 100 generations. The first 2500 trees from each run were discarded as a burn-in.

Amino acid sequences were aligned with both Clustal X [66] and MAFFT using the G-INS-i strategy [80]. BI analyses were performed with MrBayes version 3.0b4 [77,78] with the mtRev + I + G model, selected by Protest 10.2 [81] as optimal. We also implemented the CAT + GTR model in PhyloBayes 3 [82]. The ML analysis was carried out with RAxML 7.2.7 [74,75] with CAT model.

The mitochondrial genome sequences of *N*. cf. *mirabilis *and *Z. rubens *are deposited in GenBank under the accession numbers HQ997772 and HQ997773.

## Abbreviations

*atp6 *and *atp8*: ATP synthase subunits 6 and 8; *cob*: cytochrome *b*; *cox1*-*3*: subunits I-III of cytochrome *c *oxidase; *nad1*-*6 *and *nad4L*: NADH dehydrogenase subunits 1-6 and 4 L; *rrnL *and *rrnS*: the large and small subunits of ribosomal RNA; *trnX*: genes encoding for transfer RNA molecules with corresponding amino acids denoted by the one-letter code and codon indicated in parentheses (xxx) when necessary; DHU: dihydrouridine loop; MtDNA: mitochondrial DNA; NC: non-coding region; PCR: polymerase chain reaction; Kb: kilobase; bp: base pair; nt: nucleotide; nucleotide symbol combination: R = A/G; Y = C/T; W = A/T; K = G/T; N = A/G/C/T.

## Competing interests

The authors declare that they have no competing interests.

## Authors' contributions

HXC performed the majority of the molecular experiments and analyzed the data, and drafted the manuscript. SCS supervised the research. PS contributed to the analysis of the data. WCR performed part of the study, and provided technical assistance. JLN collected specimens, conceived, designed the research plan and did significant revisions of the manuscript draft. All authors read and approved the final manuscript.

## Supplementary Material

Additional file 1 **Figure S1**. Mitochondrial gene order (protein-coding genes and rRNAs only) of Nemertea and selected lophotrochozoan species and the putative bilaterian ground pattern (according to [39]). Gene segments are not drawn to scale. All genes are transcribed from left-to-right except those in gray, which are transcribed from right to left. The adjacencies *nad6/cob *and *atp8/atp6 *are underlined. The translocation of *nad2 *in the heteronemerteans and hoplonemerteans is highlight by *. Gene orders according to the following references: *Cephalothrix *[6,8], *Lineus *[7], *Paranemertes *[8], *Terebratulina *[40], *Katharina *[14], *Phoronis *[41], *Perionyx *[42], *Urechis *[43], *Sipunculus *[44]. Click here for file

Additional file 2 **Table S1**. Pairwise breakpoint distance matrix of mitochondrial gene orders of nemerteans, the bilaterian ground pattern and six other lophotrochozoans* Click here for file

Additional file 3 **Table S2**. Pairwise reversal distance matrix of mitochondrial gene orders of nemerteans, the bilaterian ground pattern and six other lophotrochozoans* Click here for file
