# Supplementary material for: A comparative study of nemertean complete mitochondrial genomes, including two new ones for Nectonemertes cf. mirabilis and Zygeupolia rubens, may elucidate the fundamental pattern for the phylum Nemertea
Source: BMC Genomics. 2012 Apr 17;13:139. doi: 10.1186/1471-2164-13-139 (PMC3368773; doi:10.1186/1471-2164-13-139)
Supplement: Additional file 1 — Figure S1. Mitochondrial gene order (protein-coding genes and rRNAs only) of Nemertea and selected lophotrochozoan species and the putative bilaterian ground pattern (according to [39]). Gene segments are not drawn to scale. All genes are transcribed from left-to-right except those in gray, which are transcribed from right to left. The adjacencies nad6/cob and atp8/atp6 are underlined. The translocation of nad2 in the heteronemerteans and hoplonemerteans is highlight by *. Gene orders according to the following references: Cephalothrix [6,8], Lineus [7], Paranemertes [8], Terebratulina [40], Katharina [14], Phoronis [41], Perionyx [42], Urechis [43], Sipunculus [44]. [file 1471-2164-13-139-S1.DOC]

Additional file 1:


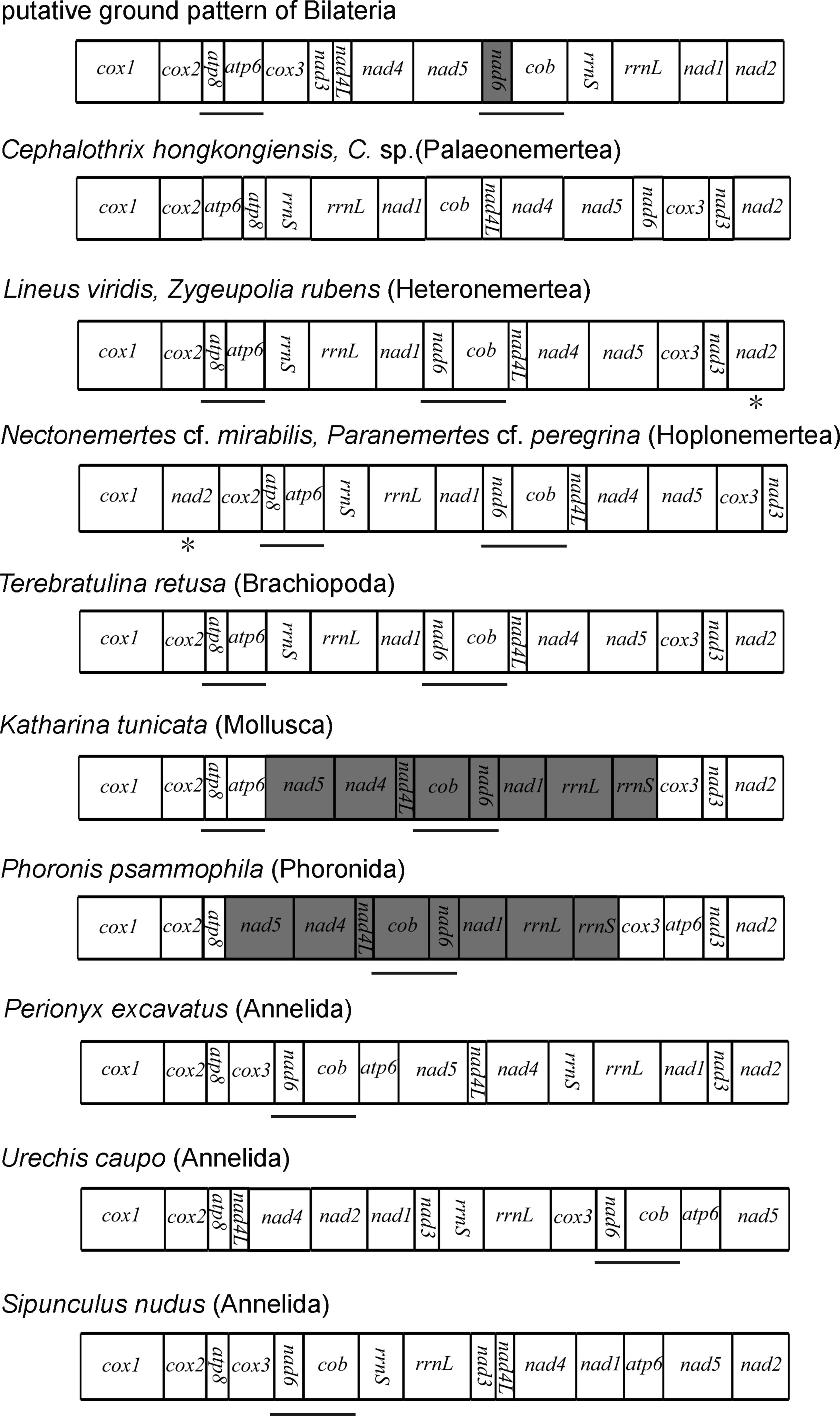


**Figure S1. Mitochondrial gene order (protein-coding genes and rRNAs only) of Nemertea and selected lophotrochozoan species and the putative bilaterian ground pattern (according to [41]).** Gene segments are not drawn to scale. All genes are transcribed from left-to-right except those in gray, which are transcribed from right to left. The adjacencies *nad6/cob* and *atp8/atp6* are underlined. The translocation of *nad2* in the heteronemerteans and hoplonemerteans is highlight by *. Gene orders according to the following references: *Cephalothrix* [6, 8], *Lineus* [7], *Paranemertes* [8], *Terebratulina* [40], *Katharina* [14], *Phoronis* [41], *Perionyx* [42], *Urechis* [43], *Sipunculus* [44].
