# Supplementary material for: A comparative study of nemertean complete mitochondrial genomes, including two new ones for Nectonemertes cf. mirabilis and Zygeupolia rubens, may elucidate the fundamental pattern for the phylum Nemertea
Source: BMC Genomics. 2012 Apr 17;13:139. doi: 10.1186/1471-2164-13-139 (PMC3368773; doi:10.1186/1471-2164-13-139)
Supplement: Additional file 3 — Table S2. Pairwise reversal distance matrix of mitochondrial gene orders of nemerteans, the bilaterian ground pattern and six other lophotrochozoans* [file 1471-2164-13-139-S3.DOC]

Additional file 3

Table S2: Pairwise reversal distance matrix of mitochondrial gene orders of nemerteans, the bilaterian ground pattern and six other lophotrochozoans*

| Reversal distance | B | P | H | H | Tr | Kt | Uc | Sn | Pe | Pp |
| --- | --- | --- | --- | --- | --- | --- | --- | --- | --- | --- |
| Bilaterian ground pattern (B) | 0\0 | 31 | 28 | 28 | 29 | 28 | 33 | 31 | 27 | 24 |
| Palaeonemertean (P) | 8 | 0\0 | 15 | 17 | 21 | 14 | 28 | 25 | 24 | 9 |
| Heteronemertean (H) | 6 | 6 | 0\0 | 3 | 20 | 15 | 29 | 21 | 20 | 9 |
| Hoplonemertean (H) | 7 | 9 | 3 | 0\0 | 18 | 13 | 29 | 21 | 18 | 6 |
| T. retusa (Tr) | 6 | 6 | 0 | 3 | 0\0 | 17 | 27 | 19 | 17 | 12 |
| K. tunicata (Kt) | 5 | 5 | 2 | 5 | 2 | 0\0 | 32 | 22 | 22 | 9 |
| U. caupo (Uc) | 9 | 11 | 7 | 9 | 7 | 8 | 0\0 | 21 | 15 | 19 |
| S. nudus (Sn) | 8 | 11 | 9 | 11 | 9 | 9 | 7 | 0\0 | 9 | 17 |
| P. excavatus (Pe) ^a^ | 8 | 9 | 5 | 8 | 5 | 6 | 5 | 5 | 0\0 | 14 |
| P. psammophila (Pp) ^b^ | 8 | 6 | 3 | 6 | 3 | 4 | 9 | 6 | 6 | 0\0 |

*bold numbers represent pairwise reversal distances between mitochondrial gene orders (37 genes in total), while italic numbers represent pairwise common interval distances between mt gene orders without tRNAs (15 genes in total).

^a^ lacks trnR.

^b^ lacks several tRNAs.
